# *The Organon* and Materia Medica Club of the Bay Cities of California

**Published:** 1896-04

**Authors:** W. E. Ledyard

**Affiliations:** Secretary


### *THE ORGANON* AND MATERIA MEDICA CLUB OF THE BAY CITIES OF CALIFORNIA.

The regular semi-monthly meeting was held at the office of Dr. A. McNeil, 784 Van Ness Avenue, San Francisco, Friday evening, October 4th, 1895.

Members present: Drs. A. McNeil, J. M. Selfridge, W. E. Ledyard, George H. Martin, M. T. Wilson, C. M. Selfridge, and M. F. Underwood.

The meeting was called to order at 8 o'clock by the President, Dr. J. M. Selfridge. The minutes of the previous meeting were read by the Secretary, and after some slight corrections by Drs. McNeil and J. M. Selfridge, were approved.

### Discussion upon Minutes.

Dr. J. M. Selfridge—I call into question a statement made by Dr. McNeil. I hold that any remedy that will cure a psoric disease is an anti-psoric remedy.

Dr. McNeil—I meant a *chronic* disease, but not necessarily a psoric one; hence I mentioned *Rhus* in chronic rheumatism.

Dr. J. M. Selfridge—All chronic diseases originate from three conditions; either psora, syphilis, or sycosis. You controvert Hahnemann by saying any chronic disease is not psoric.

Dr. McNeil—You have misunderstood Hahnemann. He says that *nearly* all chronic diseases are from psora. We have all cured chronic rheumatism with *Rhus*, and chlorosis with *Pulsatilla*.

Dr. J. M. Selfridge read Section 80 of *The Organon.*

Dr. McNeil—Why do you give *Pulsatilla* for chlorosis? It is not an anti-psoric remedy, and yet it cures this chronic condition.

Dr. J. M. Selfridge—I do not care whether Hahnemann says so or not, any remedy that cures a psoric disease is an anti-psoric remedy.

Dr. Martin was then appointed reader of the evening and commenced at Section 264 of *The Organon* and continued to 270.

DISCUSSION.

Dr. J. M. Selfridge—I cannot see why Hahnemann shakes it twice. Why cannot it be shaken one hundred times as well as twice?

Dr. Ledyard—Does he mean the 30th potency?

Dr. Martin—Yes.

Section 271 was then read.

DISCUSSION.

Dr. Martin—According to my comprehension, it is not necessary to add more alcohol, but simply to shake it.

Dr. J. M. Selfridge—Skinner says it is not potentization, but that it is merely dilution. He had a potentizer that shook it and emptied it. I do not agree with him however.

Dr. McNeil—Dunham prepared the 200th potency by mixing first one drop of the tincture with ninety-nine drops of alcohol, thus making the first dilution; this he put into stout, heavy bottles which he put into a stamp-mill bringing the stamp up and down two hundred times. Each potency was made by one shock of the stamp-mill.

Dr. Martin—In order to have a dilution there must be something to dilute it with, as there is just as much tincture there as there was before this process of potentization.

Dr. Wilson—This appertains to pharmacy.

Dr. Ledyard—It seems to me now that it was for the purpose of decreasing the strength that he diluted.

Dr. McNeil—Hahnemann's first intention was to divide the dose, and he found that by diluting with alcohol he did not weaken it; but that by a process of shaking, the potentiality of the drug was developed.

Dr. Martin read Section 269 to explain this.

Dr. J. M. Selfridge—It separates the crude substance into molecules and atoms so that they will be better able to penetrate the organism.

Section 272 was then read.

DISCUSSION.

Dr. McNeil—Some ignorant men tried to prove that Hahnemann used *Byronia* and *Rhus* in alternation, but this is not so. During the war typhus, he had 108 cases without death, and he did not alternate. Sometimes two drugs were given, but for different conditions. It cannot be proved that he alternated.

Dr. J. M. Selfridge—He declaims against it, and cautions against the use of more than one remedy.

Sections 273–276 were then read.

DISCUSSION.

Dr. McNeil—There is one point I think it best to call attention to. Very many times a patient will ask how such minute doses can be curative? I was called to a lady in the climacteric period. She presented a perfect picture of Opium poisoning, but had taken no Opium. In such cases as these, I have asked people how large a dose they would give to a patient of this kind, and they invariably say "a very small dose." This I tell them is the answer to their question regarding the minute dose. I think this patient with symptoms of Opium poisoning would have been killed by a large dose of Opium. She was morbifically sick.

Dr. J. M. Selfridge—I had a case, many years ago, of a child with inflammation of the brain and meninges, who presented a perfect picture of Opium: contracted pupils, stupor, etc. I called in Dr. Stevenson. We both remarked the symptoms of Opium poisoning. I said that I would be afraid to give the 6th potency. I gave the 200th and cured. I believe the low potency would have killed.

Dr. Ledyard—These things also show what great folly there is in the old-school treatment. It is unscientific. They often give stimulants in such cases to make the patient rally.

Dr. McNeil—My charity goes more with the allopaths in that way. A professed homœopath who gives large doses of Opium is worse than the allopath.

Sections 277 and 278 were read.

DISCUSSION.

Dr. McNeil—In this last section we find authority from Hahnemann to use the highest potencies that are made. Men who give the highest potencies to-day are blamed for leaving Hahnemann's guidance; but no one who reads this can criticise in this manner.

Section 280 was read.

DISCUSSION.

Dr. J. M. Selfridge—There is no such thing as special force. Everything can be accounted for in nature by the application of physical and chemical sciences. In other words, almost everything we see is a form of motion. Electricity is the result of motion.

Dr. Underwood—Force and matter are two distinct things.

Dr. Martin—Electricity is a form of motion; force is the result of motion.

Dr. J. M. Selfridge—Heat is the result of motion.

Dr. Martin then made a motion that the Secretary draw up resolutions of sympathy concerning the death of Dr. James E. Lilienthal, a copy to be sent to his brother, Mr. E. R. Lilienthal; seconded by Dr. Wilson and carried.

Upon motion of Dr. Wilson, seconded by Dr. McNeil, the meeting then adjourned to meet again the third Friday in October, at the office of Dr. J. M. Selfridge in Oakland, when the reading of *The Organon* would be commenced at Section 281.

W. E. LEDYARD, *Secretary.*

Reported by Eleanor F. Martin, M. D.

